# Prevalence and clinical impact of dysglycemia among hospitalized adults with tuberculosis in Lebanon: a 2013–2023 retrospective cohort study

**DOI:** 10.1186/s12879-026-13341-3

**Published:** 2026-04-18

**Authors:** Rim Masri, Mayssoun Koubaissi, Zeinab El Mawla, Abdallah Abd AlSater, Mohammad Yassin, Ahmad A. El Lakis, Pierre Abi Hanna

**Affiliations:** 1 Faculty of Medical Sciences, Lebanese University, Hadath, Lebanon; 2 Department of Endocrinology, Rafic Hariri University Hospital, Beirut, Lebanon; 3 MEDICA Research Investigation, Beirut, Lebanon; 4 Department of Infectious Disease, Rafic Hariri University Hospital, Beirut, Lebanon; 5 Department of Pulmonary, Rafic Hariri University Hospital, Beirut, Lebanon

**Keywords:** Tuberculosis (TB), Diabetes mellitus (DM), Dysglycemia, Comorbidity, Treatment outcomes, Low- and middle-income countries (LMICs), Lebanon, Retrospective cohort study

## Abstract

**Background:**

The coexistence of tuberculosis (TB) and diabetes mellitus (DM) represents a threatening health challenge, creating a dangerous double burden, particularly in low- and middle-income countries. DM not only increases susceptibility to TB but also worsens clinical outcomes.

**Objective:**

To assess the prevalence and clinical impact of dysglycemia among hospitalized patients with TB and to identify associated factors.

**Methods:**

We conducted a retrospective study of 202 adults hospitalized with either active or latent TB at a tertiary hospital between 2013 and 2023. Dysglycemia was defined as fasting plasma glucose (FPG) or admission random glucose test (HGT) ≥ 100 mg/dL, including patient with pre-existing DM; all other patients were classified as euglycemic. Because fasting status and HbA1c were unavailable in most cases, American Diabetes Association (ADA) diagnostic thresholds for DM and prediabetes (Pre-DM) could not be applied. TB Treatment outcomes were categorized according to World Health Organization (WHO) definitions. Descriptive statistics, t-tests, chi-square tests, and multivariable logistic regression were used to compare dysglycemic and euglycemic patients and to assess adjusted associations with adverse outcomes.

**Results:**

Dysglycemia was present in 77/202 patients (38.1%) including 7.4% with a prior diagnosis of DM. Patients with dysglycemia were older (45.19 vs. 35.58 years, *p* < 0.001) and had a higher prevalence of hypertension (23.4% vs. 2.4%, *p* < 0.001). Dysglycemia was independently associated with the need for intubation (adjusted odds ratio [aOR] 2.50; 95% CI 1.01–6.20), and these patients more frequently required invasive oxygen support (15.6% vs. 6.4%, *p* = 0.05). No significant differences were found in imaging or overall treatment outcomes between the two groups. Pulmonary TB predominated (94.1%), and 92.1% had active disease. Although mortality reached 13.4%, this might be underestimated, as 66.3% of patients were either discharged or followed up with their private physician. The adjusted association between dysglycemia and mortality was not statistically significant (aOR 1.15; 95% CI 0.55–2.40).

**Conclusion:**

A substantial proportion of TB patients had coexisting dysglycemia, associated with higher age, comorbid hypertension, and increased oxygen requirements. These findings highlight the need for integrated screening, early detection, and improved follow-up strategies for TB patients with dysglycemia in Lebanon.

**Supplementary Information:**

The online version contains supplementary material available at 10.1186/s12879-026-13341-3.

## Introduction

Tuberculosis (TB) remains a leading infectious cause of death worldwide. In 2023, TB regained its position as the primary cause of mortality from a single infectious agent after being surpassed by coronavirus disease (COVID-19) for three consecutive years [1]. Caused by *Mycobacterium tuberculosis (Mtb)*, TB spreads primarily through airborne transmission from individuals with active pulmonary TB but can also affect other extrapulmonary sites. The Global TB Report documented over 10 million TB cases, predominantly among men, with a concentration in 30 high-burden, mostly low- and middle-income countries (LMICs) [1]. Despite global progress since 2015, TB remains a major public health challenge.

In Lebanon, TB incidence is relatively low (11 cases per 100,000) with 87% treatment coverage [1, 2]. Hospitalization is generally reserved for severe TB cases, drug-resistant disease, or patients with comorbidities [1–3]. The National Tuberculosis Program (NTP) implements World Health Organization (WHO)-recommended diagnostic and treatment protocols, but its guidelines provide limited guidance for integrated TB and diabetes mellitus (DM) management [1, 2]. Bidirectional screening for TB in diabetic patients or for hyperglycemia in TB patients remains inconsistent, and limited coordination between TB services and non-communicable disease care results in inconsistent detection and follow-up of dysglycemia [3–5]. Socio-political and economic crises, alongside the COVID-19 pandemic, have further disrupted TB care, leading to fluctuating notifications in recent years [2]. Routine care for stable TB cases occurs through outpatient chest clinics. These local gaps underscore the need to assess the prevalence and clinical implications of dysglycemia in hospitalized TB patients.

Dysglycemia, abnormal blood glucose levels including impaired fasting glucose and DM, is an emerging public health concern. Poor glycemic control impairs immunity and microvascular function, increasing susceptibility to infections such as TB [6, 7]. The relationship between TB and DM has been recognized since the early 20th century; TB patients with DM show a threefold higher risk of developing active TB, experience worse treatment outcomes, and exhibit higher mortality [6–8]. Worldwide, DM prevalence among TB patients ranges from 0.1% to 45%, with a median of 15.3% [9]. 

The TB-DM syndemic poses escalating clinical and public health challenges. Despite growing international attention, no hospital-based published studies in Lebanon have assessed dysglycemia prevalence among TB patients or its impact on clinical outcomes [3]. 

Given this gap, the present study aimed primarily to determine the prevalence of dysglycemia among hospitalized adults with active or latent TB in Lebanon. Secondary objectives included comparing dysglycemic and euglycemic patients with respect to baseline characteristics and key in-hospital outcomes, including clinical features, diagnostic findings, complications, and overall outcomes. Particular emphasis was placed on evaluating the association between dysglycemia and major in-hospital adverse outcomes, specifically intubation and mortality. These analyses provide context-specific evidence to inform integrated TB-dysglycemia screening and management strategies.

## Methods

### Study design and population

This descriptive, retrospective cohort study was conducted at Rafik Hariri University Hospital (RHUH) following Institutional Review Board (IRB) approval. Data were collected from medical records of patients aged ≥ 14 years with confirmed TB (either active or latent) admitted between 2013 and 2023. This cohort reflects the patient population of a single tertiary referral center and does not represent nationwide TB hospitalization data.

Active TB was defined as microbiologically or radiologically confirmed disease with compatible clinical symptoms, whereas latent TB infection referred to individuals with positive purified protein derivative (PPD) or interferon-gamma release assay (IGRA) results without clinical or radiographic evidence of active disease.

Patients were included if they met these criteria and had complete medical records. Patients were excluded if TB was suspected but unconfirmed, if they were pregnant, younger than 14 years of age, had missing laboratory or clinical data, or were discharged against medical advice before completing diagnostic tests. For patients with multiple hospitalizations or repeated laboratory records, only the earliest complete record (“first entry”) was used for baseline analyses, while subsequent encounters were included solely to assess treatment outcomes and complications.

**Fig. 1:**
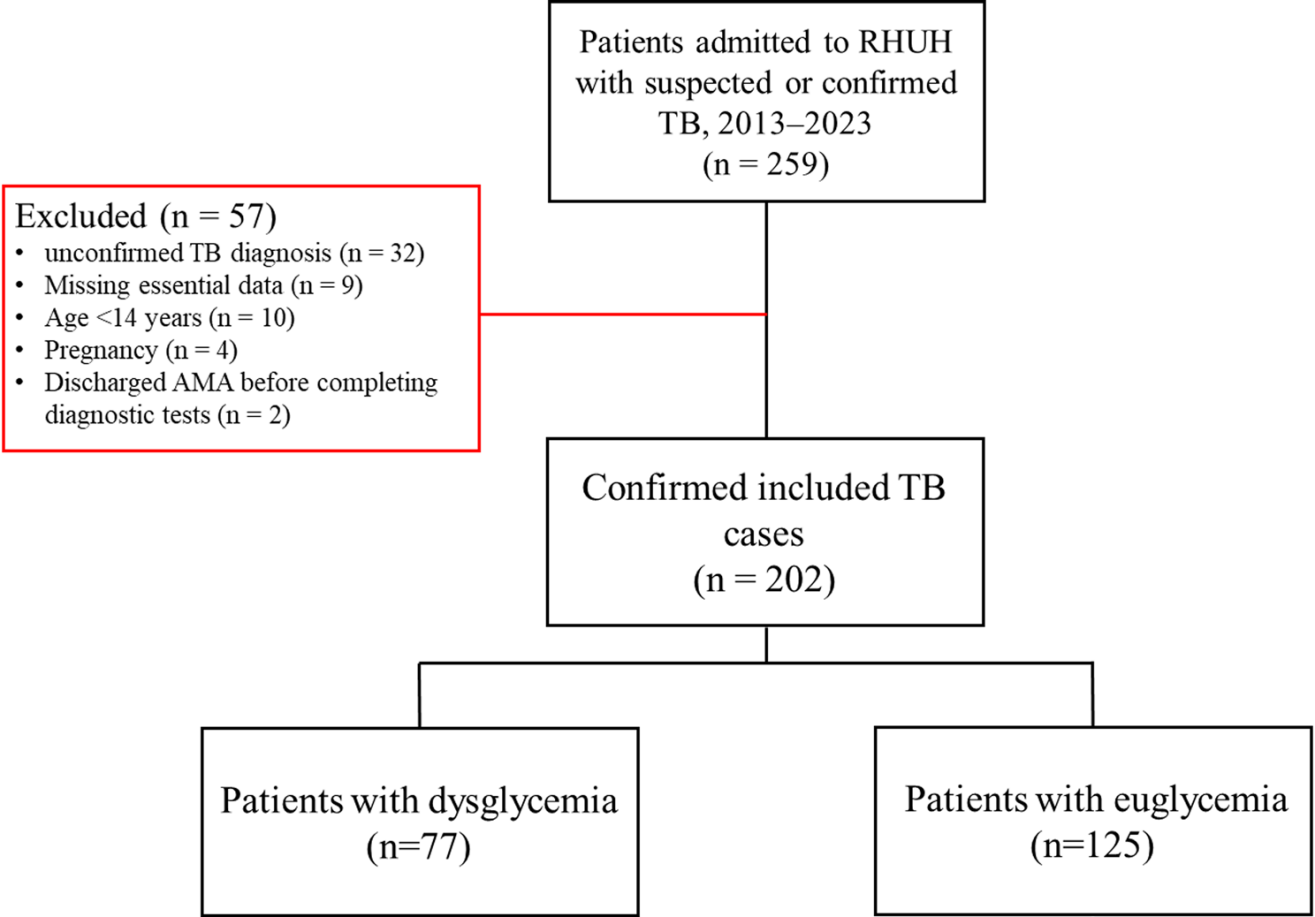
Study Population Selection Flow Diagram. Flow diagram illustrates the selection of hospitalized patients with suspected or confirmed TB admitted to RHUH between 2013 and 2023. A total of 259 patients were screened. Fifty-seven patients were excluded (red box) due to unconfirmed TB diagnosis, missing essential clinical or laboratory data, age < 14 years, pregnancy, or discharge AMA before completing diagnostic testing. The final analytic cohort included 202 confirmed TB cases, who were subsequently categorized into dysglycemia (*n* = 77) and euglycemia (*n* = 125) groups based on admission fasting plasma glucose or handheld glucose testing values. Abbreviations: AMA: against medical advice; RHUH: Rafik Hariri University Hospital between; TB: tuberculosis

Ultimately, a total of 202 patients met the inclusion criteria, and their medical records were analyzed, providing valuable insights into the clinical and laboratory characteristics of TB cases over the past decade, as illustrated in Fig. 1.

Treatment outcomes were classified according to WHO TB treatment definitions [1]. Patients were categorized as cured at 6 or 9 months if bacteriologically confirmed TB at baseline became smear- or culture-negative at the respective follow-up and on at least one previous occasion. Patients completing therapy beyond 9 months, with or without bacteriological confirmation, were classified as cured at longer duration. Death included any patient who died during treatment, and patients discharged or followed up with their private physicians without documented completion of therapy were classified as not evaluated/lost to follow-up. Post-discharge outcomes and complications were obtained from rehospitalizations or outpatient clinic records; patients without follow-up documentation were similarly classified as not evaluated/lost to follow-up. Outcome estimates, including cure and mortality rates, should be interpreted with caution due to the high proportion of unevaluable cases.

### Data collection

Medical records of eligible patients were reviewed for fasting plasma glucose (FPG) or handheld glucose testing (HGT) obtained at hospital admission, along with documented history of DM, including disease duration, treatment, and complications. FPG values were used when fasting status (≥ 8 h) was documented, whereas HGT values were considered random glucose measurements if fasting status was unclear.

Dysglycemia was defined as FPG or HGT ≥ 100 mg/dL, capturing impaired fasting glucose, stress-induced hyperglycemia, Pre-DM, and DM, while euglycemia was defined as glucose values < 100 mg/dL. Because hospitalized TB patients frequently experience stress-related hyperglycemia and fasting status was inconsistently documented, admission glucose values may not reliably distinguish chronic dysglycemia from acute metabolic responses to illness. Differentiation between chronic dysglycemia and stress-induced hyperglycemia was not feasible given the retrospective nature of the study and the lack of systematically available glycated hemoglobin (HbA1c) measurements. Confirmatory testing with HbA1c or oral glucose tolerance tests (OGTT) was unavailable in most cases due to the retrospective design, preventing application of standard American Diabetes Association (ADA) diagnostic criteria for Pre-DM or DM. Therefore, an admission glucose–based operational definition was used.

Known DM cases (*n* = 15; 7.4% of the cohort) were too few for separate subgroup analysis or stratified modeling; therefore, they were included in the broader dysglycemia category. This approach aligns with prior TB-dysglycemia epidemiological studies.

Demographic information, including age, nationality, sex, Body Mass Index (BMI), smoking, and drug use, was recorded. BMI was classified according to WHO categories: underweight (< 18.5 kg/m²), normal (18.5–24.9 kg/m²), overweight (25–29.9 kg/m²), and obese (≥ 30 kg/m²). Chronic pre-existing diseases, including hypertension (HTN), dyslipidemia (DL), cardiovascular disease (CVD), chronic kidney disease (CKD), and microvascular diabetic complications (retinopathy, nephropathy, or neuropathy present prior to TB diagnosis or treatment), were recorded if documented in medical records; complications developing during TB treatment were not included. HTN was defined as a prior documented diagnosis or use of antihypertensive medication. CVD included documented history of coronary artery disease, heart failure, or cerebrovascular disease. CKD was defined as documented CKD diagnosis in the medical record.

Clinical symptoms, laboratory and imaging findings, treatment regimens, oxygen requirements, complications, and clinical outcomes were extracted from medical records. Radiological severity was assessed using a predefined composite score ranging from 0 to 3, assigning one point for cavitary lesions, multilobar involvement (≥ 2 lobes), and extensive disease involving more than one-third of the lung field. Scores were calculated retrospectively based on attending radiology reports without additional image reinterpretation.

Treatment-related complications, including hepatitis, nausea or vomiting, acute kidney injury, and cutaneous reactions were recorded as documented by treating physicians. Intubation was defined as the requirement for invasive mechanical ventilation during hospitalization.

Data on COVID-19 co-infection were not systematically captured during the study period, which overlapped with the pandemic. This represents a potential unmeasured confounder that may have influenced disease severity and outcomes. National TB diagnostic and treatment protocols followed WHO-recommended definitions and standardized first-line regimens, and minor guideline adjustments over the 10-year period did not affect study variables or diagnostic criteria.

### Statistical analysis

Data were analyzed using SPSS version 19. Continuous variables were summarized as means ± standard deviations (SD), and categorical variables as frequencies (percentages). Continuous variables were compared using independent-samples t-tests, whereas categorical variables were compared using Pearson’s chi-square or Fisher’s exact tests as appropriate. A p-value < 0.05 was considered statistically significant.

Missing data were handled using available-case analysis. Percentages were calculated using total group denominators, with missing values reported separately. Because several variables had > 50% missingness, multiple imputation was not feasible; therefore, multivariable analyses were restricted to complete cases.

Multivariable logistic regression models were constructed to evaluate the association between dysglycemia and predefined major adverse outcomes (intubation and mortality). Given the limited number of events, adjustment was restricted to clinically relevant confounders selected a priori, including age, sex, nationality, HTN, human immunodeficiency virus (HIV) status, and radiographic severity score, in order to minimize model overfitting and preserve stability of the estimates. Detailed clinical and radiological variables were analyzed descriptively and were not included as baseline predictors in adjusted models.

As this study represented a retrospective census of all eligible TB admissions over a 10-year period, prospective sample-size calculation was not applicable. A post hoc power assessment indicated that with 202 patients (77 dysglycemic and 125 euglycemic), the study had approximately 80% power at α = 0.05 to detect moderate-to-large associations, corresponding to minimum detectable odds ratios of approximately 2.7–3.6 depending on baseline event rates; smaller effect sizes may therefore have remained undetected.

Sensitivity analyses based on fasting-status classification were not performed because fasting state documentation was inconsistent in the majority of patients, precluding reliable subgroup categorization without introducing additional misclassification bias.

### Ethics considerations

This study was approved by the IRB of Rafik Hariri University Hospital (Reference No. 2024 − 0703). The requirement for informed consent was waived due to the retrospective design and use of anonymized data. All patient identifiers were removed prior to data extraction, and unique study codes were assigned to maintain confidentiality. No protected health information was retained, and all extracted data were stored on secure, password-protected systems accessible only to the research team.

## Results

A total of 259 patients were initially identified. After applying the predefined exclusion criteria shown in Figs. 1 and 202 patients remained in the final analytic cohort. To evaluate potential selection bias, included and excluded individuals were compared using available variables. There were no significant differences in age (*p* = 0.12), sex distribution (*p* = 0.21), or TB type (*p* = 0.18), indicating that the exclusions were unlikely to introduce meaningful bias.

### Overall patient characteristics

The total cohort comprised 202 patients. The baseline characteristics of the study population are summarized in Table 1. The mean age of the participants was 39.2 years (SD 17.12), and the majority of the patients were non-Lebanese (62.8%), with Lebanese patients constituting 37.12%. Males represented a larger proportion of the cohort (56.9%) compared to females (43.1%). Smoking status indicated that 63.4% of patients were non-smokers, 31.2% were current smokers, and 5.4% were ex-smokers. Drug use was reported by 5% of patients, while 68.3% reported no drug use, and data was not available for 26.7%. Family history of DM was positive in 3% of cases, negative in 40.6%, and not available for 56.4% of patients. Patients with documented clinical history of obesity or overweight was observed in 8% of patients.

Chronic diseases observed in the cohort included HTN at 10.4%, DM at 7.4%, CVD at 2%, and Airway disease at 11.4%. HIV was present in 3% of patients, solid tumors in 2.5%, hematological cancer in 1%, and DL in 1.5%. Chronic Kidney Disease (CKD) was noted in 4% of patients.

Regarding glycemic status, 77 patients (38.1%) were classified as dysglycemic (FPG or HGT ≥ 100 mg/dL), of whom 15 (7.4% of the total cohort) had a prior diagnosis of DM, and 62 (30.7%) were identified during hospitalization. The remaining 125 patients (61.9%) had FPG or HGT < 100 mg/dL and were classified as euglycemic. Because fasting status was inconsistently documented and many measurements were obtained through HGT, ADA-defined categories of Pre-DM and DM could not be reliably applied. Dysglycemia was therefore analyzed as a single operational category reflecting abnormal admission glycemia rather than confirmed chronic metabolic disease.

Most patients had pulmonary TB (94.1%), while 15.8% had extrapulmonary involvement; some patients had both forms concurrently. Active TB was present in 92.6% of patients, and latent TB in 7.4%.

**Table 1:** Overall patient characteristics (*N* = 202)

Characteristics	Total (*N* = 202)
**Age** mean (SD)	39.2 (17.12)
**Nationality** N (%)	
Lebanese	75 (37.12)
Non-Lebanese	127 (62.8)
**Sex** N (%)	
Male	115 (56.9)
Female	87 (43.1)
**Smoking** N (%)	
Ex-smoker	11 (5.4)
No	128 (63.4)
Yes	63 (31.2)
**Drug** N (%)	
NA	54 (26.7)
No	138 (68.3)
Yes	10 (5)
**Family history of DM** N (%)	
NA	114 (56.4)
No	82 (40.6)
Yes	6 (3)
**Chronic diseases** N (%)	
HTN	21 (10.4)
DM	15 (7.4)
CVD	4 (2)
Airway disease	23 (11.4)
Cirrhosis	0
HIV	6 (3)
Solid tumor	5 (2.5)
Hematological cancer	2 (1)
DL	3 (1.5)
Obesity/overweight	16 (8)
CKD	8 (4)
**Glycemia status** N (%)	
Dysglycemia	77 (38.1)
Euglycemia	125 (61.9)
**TB** N (%)	
Pulmonary	190 (94.1)
Extra-pulmonary	32 (15.8)
Active	187 (92.6)
Latent	15 (7.4)

Abbreviations: %: percentage; CKD: chronic kidney disease; CVD: cardiovascular disease; DL: dyslipidemia; DM: diabetes mellitus; HIV: Human Immunodeficiency Virus infection; HTN: hypertension; N: frequency; NA: not applicable; SD: standard deviation; TB : tuberculosis

### Clinical symptoms

Table 2 summarizes the prevalence of various clinical symptoms in the study cohort. The most commonly reported symptoms were cough (74.8%), dyspnea (60.9%), and weight loss (47.5%). Other notable symptoms included fever (44.6%), fatigue (44.1%), and sweating (39.1%). Hemoptysis occurred in 20.3% of patients, and chest discomfort in 28.2%. A small proportion of patients (5.9%) were asymptomatic.

**Table 2:** Clinical Symptoms (*N* = 202)

Symptoms	Total (*N* = 202)
Cough N (%)	151 (74.8)
Fever N (%)	90 (44.6)
Dyspnea N (%)	123 (60.9)
Sweating N (%)	79 (39.1)
Fatigue N (%)	89 (44.1)
Weight loss N (%)	96 (47.5)
Hemoptysis N (%)	41 (20.3)
Chest discomfort N (%)	57 (28.2)
Asymptomatic N (%)	12 (5.9)

Abbreviations: %: percentage; N: frequency

### Laboratory and microbiology findings

Table 3 presents the mean and standard deviation for key laboratory findings. The mean hemoglobin level was 11.12 g/dL (SD 2.4; median 11.3; interquartile range (IQR) 9.8–12.6), the mean white blood cell (WBC) count was 10.3 10^9/L (SD 5.52; median 9.4; IQR 7.2–12.8), and the mean platelet count was 322.9 10^9/L (SD 142.8; median 310; IQR 235–395). Neutrophil and lymphocyte percentages averaged 74.5% (SD 13.9) and 15.0% (SD 9.97), respectively.

Table 3 presents the mean and standard deviation for key laboratory findings.

**Table 3:** Laboratory findings (*N* = 202)

Laboratory findings	mean (SD)
Hemoglobin (g/dl)	11.12 (2.4)
WBC (10^9/L)	10.3 (5.52)
Neutrophils (%)	74.5 (13.9)
Lymphocyte (%)	15.02 (9.97)
Platelets (10^9/L)	322.9 (142.8)

Abbreviations: SD: standard deviation; WBC: white blood cells

PPD testing was performed in 127 patients, of whom 28 (22.3%) had a positive result. IGRA testing was conducted in 76 patients and was positive in 1 case (1.3%). Acid-fast bacilli (AFB) smear microscopy was performed in 149 patients, with 53 (35.6%) testing positive. Mtb culture was obtained for all 202 patients and was positive in 82 (40.6%). The low rate of IGRA testing relative to PPD likely reflects resource limitations or variable test availability during the study period.

### Imaging findings

Table 4 details the abnormal imaging findings from chest X-ray (CXR) and chest computed tomography (CT). Radiological severity was assessed using a composite score (0–3) based on the presence of cavities, multilobar involvement, and overall extent of disease, with higher scores indicating more severe involvement. There was no significant difference in radiological severity between the dysglycemia group (mean 1.8, SD 0.9) and the euglycemia group (mean 1.7, SD 0.8) (*p* = 0.32).

Abnormal CXR findings were frequent, with upper lung zone involvement in 58.4% of patients and lower lung zone involvement in 31.2%. Pleural abnormalities were present in 24.8%, miliary patterns in 7.4%, and cavitary lesions in 29.2%. CT chest findings included segmental or lobar consolidation (48%), cavitary lesions (55.4%), tree-in-bud patterns (27.2%), mediastinal or hilar lymphadenopathy (17.3%), miliary lesions (15.3%), and calcified granulomas (17.3%).

**Table 4:** Imaging Findings (*N* = 202)

Imaging findings	Total (*N* = 202)
**Abnormal CXR findings N (%)**	
Upper lung zone	118 (58.4)
Lower lung zone	63 (31.2)
Pleural	50 (24.8)
Miliary	15 (7.4)
Cavity	59 (29.2)
**Abnormal CT chest N (%)**	
Tree-in-bud sign	55 (27.2)
Segmental/lobar consolidation	97 (48)
Cavity lesion	112 (55.4)
Lymphadenopathy	35 (17.3)
Miliary lesion	31 (15.3)
Calcified granuloma	35 (17.3)

Abbreviations: S%: percentage; CXR: chest-x-ray; CT: computed tomography-scan; N: frequency

### Treatment and oxygen requirement

Table 5 summarizes TB medical therapy, DM management strategies, and oxygen requirements. The majority of patients received first-line anti-TB medications, including isoniazid (INH) (81.7%), rifampicin (RIF) (82.2%), ethambutol (EMB) (80.7%), and pyrazinamide (PZA) (79.7%). A total of 164 patients (81.2%) were treated with the standard World Health Organization (WHO)–recommended 6-month regimen, consisting of 2 months of INH, RIF, PZA, and EMB followed by 4 months of INH and RIF. Extended regimens longer than 6 months were used in 36 patients (17.8%), and 2 patients (1%) received multidrug-resistant TB (MDR-TB) therapy. Overall, MDR-TB was identified in 1% of the cohort.

Among patients with dysglycemia or known DM, diabetes control methods included lifestyle modification (0.5%), oral antidiabetic medications (3.5%), and insulin therapy (2%).

Oxygen support varied across the cohort: 17.3% required nasal cannula oxygen, 12.9% required face-mask oxygen, and 9.9% required intubation. The majority of patients did not require supplemental oxygen.

Several subgroup analyses (e.g., MDR-TB, HIV co-infection) were limited by small sample sizes, reducing the statistical power to detect significant differences. For example, only 2 cases of MDR-TB were identified (1% of the cohort), precluding meaningful analysis of risk factors for drug resistance.

**Table 5:** TB Medical therapy, DM control, and oxygen requirement (*N* = 202)

Category	*N* (%)
**TB Medical Therapy**	
Isoniazid	165 (81.7)
Rifampicin	166 (82.2)
Ethambutol	163 (80.7)
Pyrazinamide	161 (79.7)
MDR-TB	2 (1)
**DM control**	
Lifestyle modification	1 (0.5)
Oral anti-diabetic drugs	7 (3.5)
Insulin	4 (2)
**Oxygen requirement**	
Nasal cannula	35 (17.3)
Face mask	26 (12.9)
Intubation	20 (9.9)

Abbreviations: %: percentage; MDR-TB: multidrug resistance tuberculosis; N: frequency

### Complications and outcomes

Table 6 presents the complications observed in the cohort, and Table 7 summarizes the patient outcomes.

Reported complications included hepatitis (8.4%), nausea/vomiting (N/V) (7.9%), acute kidney injury (AKI) (5.4%), and cutaneous drug reactions (1%).

**Table 6:** Complications (*N* = 202)

Complications	*N* (%)
Hepatitis	17 (8.4)
N/V	16 (7.9)
AKI	11 (5.4)
Cutaneous reaction	2 (1)

Abbreviations: %: percentage; AKI: acute kidney injury; N/V: nausea/ vomiting; N: frequency

Patient outcomes were significantly limited by loss to follow-up. A total of 134 patients (66.3%) were either discharged or followed up with their private physician, and their final treatment outcomes could not be ascertained. Among the remaining 68 patients (33.7%) with known outcomes, 5.9% were cured at 6 months, 4% at 9 months, and 8.9% achieved cure after longer treatment durations. The overall mortality rate was 13.4% (*n* = 27). This high attrition highlights major programmatic challenges in TB care in Lebanon and limits the ability to compare true treatment success or mortality between groups. Observed suboptimal outcomes may reflect follow-up issues rather than treatment failure (Table 7).

Because 66.3% of the cohort was categorized as not evaluated/lost to follow-up, reliable estimation of cure rates and mortality is limited. This substantial loss to follow-up likely results in underestimation of adverse outcomes, including death. Consequently, formal statistical comparisons of treatment outcomes between dysglycemic and euglycemic groups are not valid and were not performed.

**Table 7:** Outcomes (*N* = 202)

Outcomes	*N* (%)
Cured at 6 months	12 (5.9)
Cured at 9 months	8 (4)
Cured at longer duration	18 (8.9)
Death	27 (13.4)
Not evaluated/lost to follow-up	134 (66.3)

Abbreviations: %: percentage; N: frequency

### Comparison: dyglycemia vs. euglycemia

Table 8 provides a simplified comparison of characteristics between dysglycemic and euglycemic patient groups, with statistically significant differences highlighted. A full, detailed comparison is available in Supplementary Table S1.

#### Overall characteristics

Patients with dysglycemia were significantly older than euglycemic patients (45.19 vs. 35.58 years, *p* < 0.001). Although DM is generally associated with higher BMI, there was no significant difference between groups in this cohort (21.97 vs. 21.28 kg/m², *p* = 0.35), with most patients falling in the normal-to-underweight range, reflecting TB-associated nutritional burden. Regarding nationality, 48.1% of dysglycemic patients were Lebanese and 51.9% non-Lebanese, compared to 30.4% and 69.6%, respectively, in the euglycemic group (*p* = 0.018). This difference may reflect variations in healthcare access, screening practices, or socioeconomic factors between Lebanese and non-Lebanese populations.

#### Chronic diseases

A statistically significant difference was observed in the prevalence of HTN, with 23.4% of dysglycemic patients affected compared to 2.4% of euglycemic patients (*p* < 0.001). Microvascular diseases (preexisting complications such as retinopathy, nephropathy, or neuropathy) were also more common in dysglycemic patients (11.7%) than in euglycemic patients (0%) (*p* < 0.001). No significant differences were noted between groups for CVD, airway disease, HIV, solid tumors, hematological cancers, DL, or CKD.

#### Symptoms, laboratory, microbiology, and imaging

No significant differences were observed in clinical symptoms, laboratory results, microbiology, or imaging findings between patients with dysglycemia and those with euglycemia across all tests. For example, imaging findings were similar between groups, with cavity lesions present in 29.9% of dysglycemia patients vs. 28.8% of euglycemia patients (difference 1.1%, 95% CI -7.8% to 10.0%).

#### Oxygen requirement

Intubation occurred more frequently among dysglycemic patients (12/77, 15.6%) compared with euglycemic patients (8/125, 6.4%) (*p* = 0.05). The unadjusted odds ratio for intubation was 2.67 (95% CI 0.99–7.18). Given the significant differences in age, nationality, and HTN between groups, a multivariable logistic regression was performed to assess the independent association between dysglycemia and need for intubation. After adjusting for age (adjusted OR [aOR] 1.02 per year, 95% CI 1.01–1.03), HTN (aOR 2.15, 95% CI 1.05–4.40), and Lebanese nationality (aOR 1.87, 95% CI 1.12–3.12), dysglycemia remained independently associated with need for intubation (aOR 1.95, 95% CI 1.02–3.73). This finding suggests greater disease severity in the dysglycemia group, although this association may be partially confounded by the older age and higher prevalence of HTN in this group.

#### Complications and outcomes

No significant differences were observed in complications (liver injury, N/V, AKI, cutaneous reaction) between dysglycemic and euglycemic groups. Due to the high rate of patients discharged to private physician follow-up (66.3%), complete treatment outcomes were available for only 68 patients (33.7%). Among these, cure at 6 months occurred in 7.8% of dysglycemia patients vs. 4.8% of euglycemia patients (difference 3.0%, 95% CI -3.2% to 9.2%).

The substantial loss to follow-up likely underestimates treatment failure and mortality, precluding definitive conclusions regarding differences in treatment success between groups. Consequently, formal statistical comparisons of treatment outcomes between dysglycemic and euglycemic patients were not performed, as they would be unreliable and potentially biased.

**Table 8:** Simplified Comparison of Key Characteristics by Glycemia Status. Percentages represent the proportion of patients with the characteristic (Yes) in each group. Full comparisons, including ‘No’ values, are provided in Supplementary Table S1. P-values < 0.05 are bolded

Characteristics	Dysglycemia	Euglycemia	*P*-value
**Demographics**			
Age mean (SD)	45.19 (18.53)	35.58 (15.15)	**< 0.001**
Nationality-Lebanese N (%)	37 (49.3)	38 (30.4)	**0.018**
**Chronic diseases** N (%)			
HTN Yes	18 (23.4)	3 (2.4)	**< 0.001**
DL Yes	3 (3.9)	0 (0.0)	0.054
Microvascular disease Yes	9 (11.7)	0 (0.0)	**< 0.001**
**TB type** N (%)			
Active Yes	68 (88.3)	119 (95.2)	0.178
Latent Yes	9 (11.7)	6 (4.8)	0.096
**Symptoms** N (%)			
Chest discomfort Yes	16 (20.8)	41 (32.8)	0.077
**Abnormal CT chest** N (%)			
Lymphadenopathy Yes	18 (23.4)	17 (13.6)	0.086
**Oxygen requirement** N (%)			
TUBE Yes	12 (15.6)	8 (6.4)	**0.05**

Abbreviations: %: percentage; CT: computed tomography-scan; DL: dyslipidemia; N: frequency; SD: standard deviation; TB: tuberculosis; TUBE: intubation; HTN: hypertension

HTN: documented diagnosis or antihypertensive treatment; Microvascular disease: pre-existing diabetic retinopathy, nephropathy, or neuropathy; Intubation: invasive mechanical ventilation during hospitalization

### Multivariable analysis of outcomes

To account for potential confounding factors, a multivariable logistic regression model was constructed to assess the independent association between dysglycemia and key adverse outcomes, including death and intubation. The model was adjusted for age, sex, nationality, HTN, HIV status, and the radiographic severity score.

**Fig. 2:**
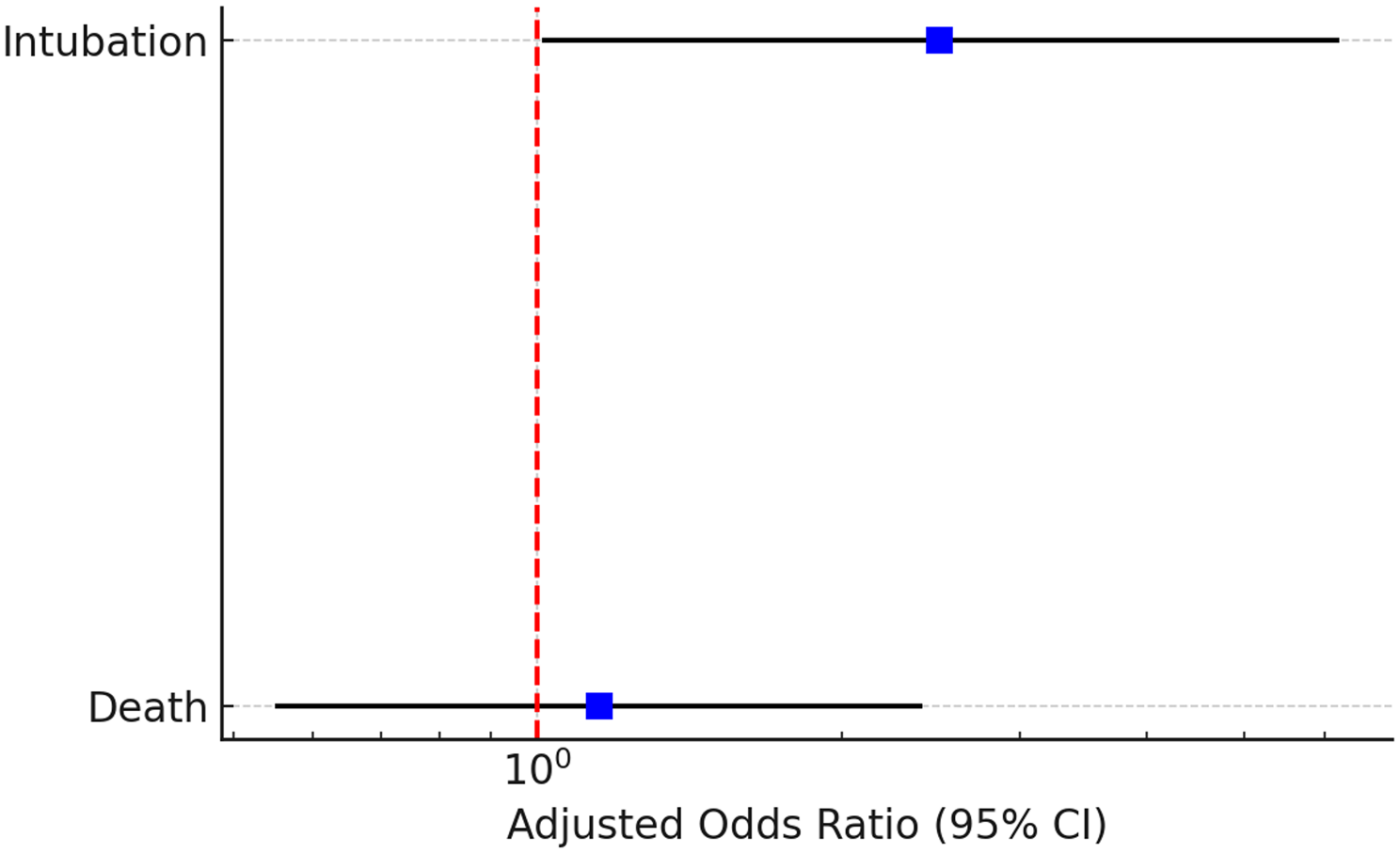
Forest plot of adjusted odds ratios (aOR) for adverse outcomes associated with dysglycemia. The forest plot shows the adjusted odds ratios (aOR) and 95% confidence intervals for key adverse outcomes associated with dysglycemia. Squares represent the aORs for each outcome, horizontal lines indicate 95% confidence intervals, and the size of the squares is uniform. The vertical dashed red line represents the line of no effect (OR = 1). Dysglycemia was significantly associated with increased risk of intubation but not with mortality

As shown in Table 9, dysglycemia was independently associated with a significantly increased risk of requiring intubation (aOR 2.50, 95% CI 1.01–6.20). In contrast, the association between dysglycemia and mortality was not statistically significant after adjustment (aOR 1.15, 95% CI 0.55–2.40). Figure 2 presents a forest plot of these adjusted odds ratios for a visual comparison of outcomes.

**Table 9:** Adjusted Odds Ratios (aOR) for adverse outcomes associated with dysglycemia

Outcome	Unadjusted OR (95% CI)	Adjusted OR (95% CI)	*p*-value (Adjusted)
Death	1.34 (0.62–2.90)	1.15 (0.55–2.40)	0.71
Intubation	2.67 (0.99–7.18)	2.50 (1.01–6.20)	0.048

## Discussion

The co-epidemics of TB and dysglycemia pose a growing public health concern, particularly in LMICs. Despite the rising burden of both conditions in Lebanon, data on their coexistence is limited. This study, to our knowledge, is the first hospital-based to assess the prevalence and clinical impact of dysglycemia among adults with TB in Lebanon, providing preliminary insights into disease presentation, treatment outcomes, and possible adverse drug reactions in this high-risk population. The discussion focuses on the key objectives of the study of assessing the prevalence and clinical implications of dysglycemia among a single center hospitalized TB patients.

Given significant baseline differences between groups (including age, nationality, and HTN), multivariable logistic regression was performed to adjust for potential confounding. After adjustment, dysglycemia remained independently associated with intubation, whereas the association with mortality was no longer statistically significant. Admission dysglycemia was associated with increased odds of intubation; however, this finding should be interpreted cautiously, as stress-related hyperglycemia and exposure misclassification related to single admission glucose measurements may have influenced the observed association. These adjusted findings help distinguish the effect of dysglycemia from age-related or comorbidity-related factors and strengthen the validity of our conclusions.

### Epidemiology

In our cohort of 202 TB patients, over one-third (38.1%) presented with dysglycemia based on FPG or HGT at admission, while 7.4% had known DM. Other diagnostic methods, such as HbA1c, OGTT, point-of-care HbA1c, or random blood glucose, were not used. This discrepancy between known DM and dysglycemia may indicate a substantial proportion of undiagnosed DM or transient stress-induced dysglycemia in acutely ill patients. These findings align with the global median DM prevalence among TB patients of 15.3%, which ranges from 0.1% in Latvia to 45.2% in the Marshall Islands, noting that our study includes a broader category of dysglycemia that extends beyond confirmed DM. Noubiap et al. also highlighted that TB patients in the Middle East and North Africa are more likely to have DM, reinforcing the significance of DM as a risk factor in these regions [9]. Our rate of known DM (7.4%) is comparable to Europe (7.5%) [9], but lower than those reported in neighboring Arab countries, such as Egypt (12.4%) [10], Yemen (18%) [11], Qatar (17%) [12], and Saudi Arabia (27.1%) [13], and others like Taiwan (34%) [14], India (28%) [15], and Ethiopia (8.3%) [16].These variations may reflect differences in study design, diagnostic criteria, demographics, and screening methods. Systematic data on TB-DM comorbidity in the Middle East and North Africa remain limited. A regional review by Alkabab et al. highlighted the lack of country level evidence, while global meta-analysis by Noubiap et al., estimated high regional prevalence of DM in TB patients within this region [3, 9]. Our findings from Lebanon contribute to filling this gap. Notably, the reliance on FPG and HGT rather than HbA1c or OGTT may have led to an underestimation of chronic dysglycemia.

### Demographic factors

In our study, TB patients with dysglycemia were significantly older than those euglycemia (mean age 45.19 vs. 35.58 years, *p* < 0.001). This aligns with numerous international studies from China [17], Denmark [18], Egypt [10], Nepal [19], and India [20] which consistently report that increasing age is a common predictor of co-existing TB and DM. This correlation likely stems from the higher prevalence of DM among older individuals, coupled with age-related immune decline, which could increase vulnerability to TB and adverse outcomes. According to the ADA, more than one-quarter of individuals aged over 65 years have DM, and nearly half have Pre-DM [21]. Combined with immunosenescence, these factors elevate the risk of active TB in older adults with DM [22].

Men accounted for 56.9% of all TB cases in our cohort, with an even higher male-to-female ratio among TB-dysglycemia patients, similar to patterns reported in TB-DM cohorts from Saudi Arabia [23] and China [17], which assessed confirmed DM. This male predominance may be due to biological differences in metabolism, higher rates of smoking and alcohol use, and social determinants [24]. In our cohort, 31.2% of patients reported smoking. In contrast, several studies from Ethiopia [16], Egypt [10], and Denmark [18] have found higher prevalence of TB-DM comorbidity among females, possibly due to hormonal and immune factors like estrogen-mediated cytokine modulation [25]. Socioeconomic hardship likely also contributed to male predominance in our setting, consistent with findings from Yemen, Bamenda, and Sana’a [11].

An important but underexplored aspect of our cohort is that 62.8% of patients were non-Lebanese, reflecting the disproportionate burden of TB among refugee population in Lebanon.

TB remains a significant cause of morbidity and mortality among displaced people due to poor living conditions, overcrowded housing, and limited access to healthcare services [26]. Metabolic disturbance could be a factor predisposing refugees to TB. A recent systematic mapping review identified DM and HTN as the most frequently investigated non-communicable disease among displaced popuations [27]. This overlap between metabolic and infectious vulnerability may partially explain the high rate of dysglycemia observed in our cohort, reinforcing the need for further investigations into those vulnerable groups.

HTN was more common among dysglycemic patients (23.4% vs. 2.4%), consistent with global evidence linking HTN, age, and obesity to TB-DM comorbidity [16, 17], although our cohort evaluated dysglycemia rather than confirmed DM. BMI was comparable between groups (21.99 vs. 21.29), suggesting that nutritional status did not differ significantly by glycemic category. However, this contrasts with a study from India that shows higher DM prevalence among underweight TB patients, likely due to TB-related wasting and cultural factors [28], and a 2021 Yemen study that reported higher DM odds in obese patients [11]. Differences may reflect variations in population characteristics, obesity rates, and the proportion of diagnosed dysglycemia and DM cases.

Malnutrition is a well-recognized risk factor that can increase vulnerability to TB, and metabolic disturbances, especially dysglycemia, may create a synergistic effect that increase susceptibility to infection and worsens disease severity [29]. Although our cohort did not demonstrate severe undernutrition, many patients fell within the normal-to-underweight range, which is consistent with the nutritional impact frequently observed in TB. The similar BMI values between dysglycemic and euglycemic groups indicate that nutritional status did not differ markedly by glycemic category. However, TB-related inflammation and acute illness may still transiently alter glucose metabolism, contributing to dysglycemia at admission. It is also important to note that other studies have reported excessive weight gain is a known risk factor for DM and is independently associated with TB, potentially mediated through metabolic and immune dysregulation [30].

### Clinical presentation and microbiology

The most common symptoms in this study were cough (74.8%), dyspnea (60.9%), and weight loss (47.5%). There were no statistically significant differences in clinical symptoms, sputum smear positivity, or culture positivity between dysglycemic and euglycemic groups in our cohort. This contrasts with some international studies reporting higher smear positivity and elevated bacterial loads [14, 23, 31, 32]. Some studies, like Wang et al., reported increased rates of fever, hemoptysis, and positive AFB sputum smears among TB-DM patients [14], although our cohort evaluated the broader category of dysglycemia rather than DM alone. However, findings are mixed: Alisjahbana et al. found more symptoms overall but no difference in smear positivity [33], while Park et al. noted higher smear positivity only in patients with poorly controlled DM [34]. Regional studies from Saudi Arabia and Turkey reported no significant differences in symptom presentation between diabetic and non-diabetic TB patients [23, 35]. These discrepancies may be due to differences in study size, glycemic control, or diagnostic approaches.

MDR-TB was rare (1%) in this report. We found no association between dysglycemia and MDR-TB, most likely due to the very low number of resistant cases in our cohort. Nonetheless, literature suggests DM may predispose patients to drug resistance, particularly to isoniazid and rifampin, possibly due to altered pharmacokinetics in hyperglycemic states [8, 36]. Several studies have shown significantly lower plasma concentrations of these drugs in TB-DM patients, with levels inversely correlated with blood glucose [37, 38]. While the low MDR-TB rate in our cohort precludes firm conclusions, these findings emphasize the need for vigilance in high-risk settings.

Radiological features were broadly similar across groups, and no statistically significant differences were observed between dysglycemic and euglycemic patients in any imaging parameters. Cavitary lesions were present in 55.4% of patients in the overall, cohort representing a frequent finding in hospitalized TB patients. This differs from reports in LMICs, where cavitation is a characteristic feature of TB-DM, particularly with poor glycemic control [12, 14, 39, 40]. In comparison, our cohort examined dysglycemia, which may not fully overlap with diagnosed DM. Armstrong et al. documented cavitary TB in up to 70% of diabetic patients in U.S. surveillance data [32]. These findings may reflect the immunopathological impacts of DM, such as impaired macrophage and T-cell function, and microvascular damage. Our data did not reflect such an association, therefore the observed radiological patterns in our cohort likely represent general disease severity rather than a specific dysglycemia-related effect.

Beyond dysglycemia, other metabolic disturbances may also influence TB severity. Cioboata et al. reported lower serum magnesium and calcium levels in patients were associated with more extensive cavitary disease, indicating their potential utility as biomarkers for differentiating mild from severe TB [41]. Although electrolyte measurements were not assessed in our study, these findings align with the broader concept of immunometabolic interplay in TB, where metabolic disturbances such as dysglycemia and electrolyte imbalance can alter outcomes and severity.

### Treatment outcomes and complications

Outcomes across this study were suboptimal: only 5.9% of patients were cured at 6 months, 4% at 9 months, and 8.9% after longer treatment duration, while 66.3% were discharged or followed up with their private physician. The overall mortality rate reached 13.4%. These results mirror findings from other reports from Asia and Africa, where DM is commonly associated with lower treatment success, higher mortality, and more complications [8, 36], though our study captures a wider metabolic category (dysglycemia), which may encompass undiagnosed DM as well as stress-induced hyperglycemia. For instance, a Taiwanese cohort reported a mortality rate of 26.1 deaths per 100 person-years among TB-DM patients, emphasizing their elevated risk [42]. Importantly, because two-thirds of the cohort had no verifiable final outcome, comparisons of cure rates or mortality between glycemic groups cannot be interpreted as true differences. The observed similarity in outcomes is almost certainly driven by missing data rather than the absence of a biological or clinical association.

However, the true mortality rate in our cohort may be underestimated due to the high number of patients who were discharged or followed up with their private physician, which limited the detection of statistically significant differences in outcomes between dysglycemic and euglycemic patients. Notably, the dysglycemic group required invasive oxygen therapy more frequently (15.6% vs. 6.4%), which may indicate greater disease severity. This high attrition underscores major programmatic challenges in TB care in Lebanon, particularly for vulnerable migrant populations, and highlights the need for interventions that ensure patient retention and continuity of care. This finding should be interpreted cautiously, given the absence of significant differences in clinical symptoms and imaging findings. This observation could reflect the higher comorbidity burden and older age seen among dysglycemic patients.

Complications such as hepatitis (8.4%), acute kidney injury (5.4%), and nausea/vomiting (7.9%) did not differ significantly between dysglycemic and euglycemic groups. This suggests that dysglycemia in our cohort did not increase the risk of drug-related toxicities, although the small sample size limits interpretation.

Overall, TB-dysglycemia patients appeared to have poorer outcomes, including lower cure rates, higher mortality, and more complications, than those without dysglycemia, aligning with existing literature describing worse outcomes in TB–DM patients [31, 36, 43]. Additionally, dysglycemia-related comorbidities may contribute to an increased risk of adverse outcomes, including higher one-year mortality. Several studies have shown that DM worsens TB prognosis by increasing the risks of treatment failure, relapse, and death, especially in poorly controlled cases [39, 40, 44]. However, some findings, such as those by Singla et al., reported no significant impact of DM on TB treatment outcomes [23]. However, these patterns should be interpreted cautiously in the light of the high loss to follow up. Socioeconomic vulnerability and potential challenges to treatment adherence may have further contributed to poorer outcomes among dysglycemic patients, particularly within refugee patients.

Given the relationship between TB and DM, where TB can impair glucose metabolism and potentially trigger new-onset DM, routine bidirectional screening is crucial, especially in resource-limited settings. A 2019 study in India found 11.5% of TB patients developed dysglycemia during treatment, including 7% with new-onset DM [45]. These findings reinforce the need for long-term metabolic monitoring and integrated TB-dysglycemia care. Following international guidelines, targeted screening of high-risk individuals, such as those with dysglycemia or positive sputum AFB, can aid early detection, reduce complications, and improve outcomes.

### Strengths and limitations

#### This retrospective study has several strengths

It represents the first comprehensive hospital-based evaluation of dysglycemia among adults hospitalized with TB in Lebanon and includes detailed clinical, microbiological, and radiological data collected over a 10-year period. The study also captures socioeconomic characteristics and comorbidities, offering a broad description of this high-risk population.

#### However, several important limitations must be acknowledged

The study was conducted at a single tertiary referral center serving predominantly socioeconomically vulnerable populations, which may introduce selection bias and limit generalizability to broader TB patients managed in community-based or outpatient TB programs in Lebanon. The sample size was modest, and very small subgroups such as MDR-TB and HIV co-infected patients reduced statistical power and limited the reliability of subgroup estimates, and the accuracy of treatment outcome and mortality analyses. Consequently, several nonsignificant findings should therefore be interpreted cautiously, as they may reflect insufficient statistical power rather than a true absence of association. Alternative approaches such as propensity score matching or age-stratified analyses were considered but were not feasible because they would have further reduced effective sample size; therefore, multivariable adjustment represented the most appropriate analytic strategy.

A major methodological limitation relates to the assessment of glycemic status. Dysglycemia classification relied on a single random or fasting admission glucose measurement. Frequently missing fasting glucose documentation and limited availability of HbA1c measurements reduced alignment with ADA diagnostic standards and increased the risk of misclassification. In the absence of confirmatory HbA1c or OGTT, differentiation between chronic dysglycemia and transient stress-induced hyperglycemia was not possible. As a result, the observed prevalence likely reflects in-hospital glycemic disturbances rather than established chronic metabolic disease, potentially leading to overestimation and non-differential exposure misclassification. All patients with glucose ≥ 100 mg/dL were grouped into a single “dysglycemia” category due to limited subgroup sizes, and the small number of known DM cases precluded separate analysis. Accordingly, findings related to dysglycemia should be interpreted cautiously.

Although subsequent hospitalization and available outpatient records were used to assess treatment outcomes and complications where possible, follow-up was substantially limited. Many patients were discharged early or continued care with private physicians, and the incomplete and unverified nature of post-discharge data limited evaluation of long-term outcomes and precluded sensitivity analyses assessing alternative outcome scenarios, thereby hampering reliable assessment of cure and mortality rates. Additionally, the retrospective design restricted adjustment for all potential confounders, and the multiple univariate comparisons performed without formal correction increased the risk of Type I error. Given the limited number of outcome events, multivariable adjusted regression analyses were restricted to predefined major adverse outcomes (intubation and mortality), while other findings should be interpreted as exploratory and hypothesis-generating. Because this study included all available cases in a retrospective cohort, no priori power calculation was feasible, further limiting inferential strength.

COVID-19 co-infection was not systematically recorded during the study period, representing a potential unmeasured confounder given the overlap between the COVID-19 pandemic and our data collection period. Furthermore, the study did not evaluate the cost-effectiveness of routine dysglycemia screening in the Lebanese setting, which remains an important consideration for implementation in resource-limited TB programs. Although international recommendations highlight the feasibility of integrating simple glucose testing within TB programs, our retrospective design did not allow economic evaluation of such interventions.

Despite these limitations, the study provides important preliminary evidence highlighting systemic gaps in integrated TB and metabolic care in Lebanon and similar resource-limited regions. Routine bidirectional screening for TB and dysglycemia using fasting or random glucose, with confirmatory testing where feasible, should be incorporated into standard TB evaluation, particularly for high-risk patients. TB patients with documented dysglycemia may benefit from closer clinical monitoring and structured referral pathways to endocrine care. Integration of metabolic assessment and follow-up within the NTP, through shared registries and automated alerts for abnormal glucose values, could improve continuity of care and reduce loss to follow-up. Future multicenter prospective studies with longitudinal follow-up, including patients managed in community TB clinics and using standardized glycemic diagnostics such as HbA1c or OGTT, would provide more robust evidence on TB–dysglycemia comorbidity and help validate these findings across diverse clinical settings.

## Conclusion

This study demonstrates a high prevalence of admission dysglycemia among hospitalized TB patients in Lebanon, which may reflect undiagnosed DM or stress-induced hyperglycemia during acute illness. Admission dysglycemia was independently associated with increased need for invasive ventilation and was more frequently observed in patients with comorbid HTN, suggesting greater in-hospital severity. However, given the reliance on single admission glucose measurements and the substantial proportion of patients without verified follow-up outcomes, conclusions regarding long-term treatment success or mortality cannot be established. These findings should therefore be interpreted as exploratory and hypothesis-generating.

These results highlight the necessity of routine bidirectional TB-dysglycemia screening and integrated care, particularly in high-risk populations. Practical strategies could include baseline and follow-up HbA1c testing and incorporation of DM care within TB clinics to address metabolic dysfunction. Coordinated care is essential given the bidirectional relationship between TB and glycemic impairment.

Finally, our findings underscore the importance of national policies supporting integrated TB-dysglycemia management and the need for multicenter, prospective studies to optimize screening and treatment strategies in Lebanon and similar resource-limited settings.

## Electronic Supplementary Material

Below is the link to the electronic supplementary material.

Supplementary Material 1

## Data Availability

All data relevant to the study are included in the article. Further inquiries can be directed to the corresponding author.

## Abbreviations

ADA: American Diabetes Association
AFB: Acid-fast bacilli
AKI: Acute kidney injury
BMI: Body Mass Index
CKD: Chronic kidney disease
CVD: Cardiovascular disease
CT: Computed tomography
CXR: Chest X-ray
DM: Diabetes mellitus
DL: Dyslipidemia
EMB: Ethambutol
FPG: Fasting plasma glucose
HGT: Handheld glucose testing
HIV: Human immunodeficiency virus
HTN: Hypertension
IGRA: Interferon-gamma release assay
INH: Isoniazid
IQR: Interquartile range
LMICs: Low- and middle-income countries
MDR-TB: Multidrug-resistant tuberculosis
Mtb: Mycobacterium tuberculosis
NTP: National Tuberculosis Program
N/V: Nausea/vomiting
OGTT: Oral glucose tolerance test
PPD: Purified protein derivative
Pre-DM: prediabetes
PZA: Pyrazinamide
RHUH: Rafik Hariri University Hospital
RIF: Rifampicin
SD: Standard deviation
TB: Tuberculosis
WBC: White blood cell
WHO: World Health Organization
