# Supplementary material for: Prevalence and clinical impact of dysglycemia among hospitalized adults with tuberculosis in Lebanon: a 2013–2023 retrospective cohort study
Source: BMC Infect Dis. 2026 Apr 18;26:1054. doi: 10.1186/s12879-026-13341-3 (PMC13224518; doi:10.1186/s12879-026-13341-3)
Supplement: Supplementary file 1 — Supplementary Material 1 [file 12879_2026_13341_MOESM1_ESM.docx]

**Supplementary material**

Table S1. Comparison of Characteristics by Glycemia Status**.** Abbreviations**:** %: percentage; AFB: acid fast bacilli; AKI: acute kidney injury; BMI: body mass index; CKD: chronic kidney disease; CVD: cardiovascular disease; CT: computed tomography-scan; CXR: chest x-ray; DL: dyslipidemia; DM: diabetes mellitus; FM: face mask; HIV: human immunodeficiency virus; IGRA: interferon-gamma release assay; Mtb: mycobacterium tuberculosis; N: frequency; NC: nasal cannula; N/V: nausea and vomiting; PPD: purified protein derivative; SD: standard deviation; TB: tuberculosis; TUBE: intubation; HTN: hypertension.

| **Characteristics** | **Dysglycemia** | **Euglycemia** | **P-value** |
| --- | --- | --- | --- |
| Age mean (SD) | 45.19 (18.53) | 35.58 (15.15) | <0.001 |
| **Nationality** N (%) | | | |
| Lebanese | 37 (49.3) | 38 (30.4) | 0.018 |
| Non-Lebanese | 40 (51.9) | 87 (69.6) |  |
| **Sex N (%)** | | | |
| Male | 43 (55.8) | 72 (57.6) | 0.81 |
| Female | 34 (44.2) | 53 (42.4) |  |
| **BMI mean (SD)** | 21.97 (3.78) | 21.28 (4.65) | 0.36 |
| **Smoking N (%)** | | | |
| Ex-smoker | 1. (7.8) | 5 (4) | 0.27 |
| No | 44 (57.1) | 84 (67.2) |  |
| Yes | 27 (35.1) | 36 (28.8) |  |
| **Drug N (%)** | | | |
| No | 49 (63.6) | 89 (71.2) | 0.52 |
| Yes | 5 (6.5) | 5 (4.0) |  |
| NA | 23 (29.9) | 31 (24.8) |  |
| **Family history of DM N (%)** | | | |
| No | 29 (37.7) | 54 (43.2) | 0.20 |
| Yes | 4 (5.2) | 2 (1.6) |  |
| NA | 44 (57.1) | 69 (55.2) |  |
| **Chronic diseases N (%)** | | | |
| HTN No | 59 (76.6) | 122 (97.6) | <0.001 |
| HTN Yes | 18 (23.4) | 3 (2.4) |  |
| CVD No | 74 (96.1) | 124 (99.2) | 0.3 |
| CVD Yes | 3 (3.9) | 1 (0.8) |  |
| Airway disease No | 69 (89.6) | 110 (88) | 0.822 |
| Airway disease Yes | 8 (10.4) | 15 (12) |  |
| HIV No | 76 (98.7) | 121 (96.8) | 0.651 |
| HIV Yes | 1 (1.3) | 4 (3.2) |  |
| Solid tumors No | 74 (96.1) | 123 (98.4) | 0.371 |
| Solid tumors Yes | 3 (3.9) | 2 (1.6) |  |
| Hematological cancers No | 75 (97.4) | 125 (100) | 0.144 |
| Hematological cancers Yes | 2 (2.6) | 0 |  |
| DL No | 74 (96.1) | 125 (100) | 0.054 |
| DL Yes | 3 (3.9) | 0 |  |
| CKD No | 74 (96.1) | 120 (96) | 0.639 |
| CKD Yes | 3 (3.9) | 5 (4) |  |
| Microvascular disease No | 68 (88.3) | 125 (100) | <0.001 |
| Microvascular disease Yes | 9 (11.7) | 0 |  |
| **TB N (%)** | | | |
| Pulmonary No | 3 (3.9) | 9 (7.2) | 0.380 |
| Pulmonary yes | 74 (96.1) | 116 (92.8) |  |
| Extra-pulmonary No | 67 (87) | 103 (82.4) | 0.433 |
| Extra-pulmonary Yes | 10 (13) | 22 (17.6) |  |
| Active No | 9 (11.7) | 6 (4.8) | 0.178 |
| Active yes | 68 (88.3) | 119 (95.2) |  |
| Latent No | 68 (88.3) | 119 (95.2) | 0.096 |
| Latent Yes | 9 (11.7) | 6 (4.8) |  |
| **Symptoms N (%)** | | | |
| Cough No | 22 (28.6) | 29 (23.2) | 0.409 |
| Cough Yes | 55 (71.4) | 96 (76.8) |  |
| Fever No | 44 (57.1) | 68 (54.4) | 0.771 |
| Fever Yes | 33 (42.9) | 57 (45.6) |  |
| Dyspnea No | 29 (37.7) | 50 (40) | 0.768 |
| Dyspnea Yes | 48 (62.3) | 75 (60) |  |
| Sweating No | 48 (62.3) | 75 (60) | 0.768 |
| Sweating Yes | 29 (37.7) | 50 (40) |  |
| Fatigue No | 47 (61) | 67 (53.6) | 0.311 |
| Fatigue Yes | 30 (39) | 58 (46.4) |  |
| Weight loss No | 44 (57.1) | 62 (49.6) | 0.369 |
| Weight loss Yes | 33 (42.9) | 63 (50.4) |  |
| hemoptysis No | 64 (83.1) | 97 (77.6) | 0.373 |
| hemoptysis Yes | 13 (16.9) | 28 (22.4) |  |
| Chest discomfort No | 61 (79.2) | 84 (67.2) | 0.077 |
| Chest discomfort Yes | 16 (20.8) | 41 (32.8) |  |
| Asymptomatic No | 70 (90.9) | 120 (96) | 0.218 |
| Asymptomatic Yes | 7 (9.1) | 5 (4) |  |
| **Microbiology** | | | |
| PPD (positive) N (%) No | 62 (80.5) | 95 (76) | 0.45 |
| PPD (positive) N (%) Yes | 15 (19.5) | 30 (24) |  |
| IGRA (positive) N (%) no | 76 (98.7) | 124 (99.2) | 1 |
| IGRA (positive) N (%) Yes | 1 (1.3) | 1 (0.8) |  |
| AFB (positive) N (%) No | 60 (77.9) | 89 (71.2) | 0.29 |
| AFB (positive) N (%) Yes | 17 (22.1) | 36 (28.8) |  |
| Mtb Culture (positive) N (%) No | 42 (54.5) | 78 (62.4) | 0.28 |
| Mtb Culture (positive) N (%) Yes | 35 (45.5) | 47 (37.6) |  |
| **Abnormal CXR findings N (%)** | | | |
| Upper lung zone No | 29 (37.7) | 55 (44) | 0.383 |
| Upper lung zone Yes | 48 (62.3) | 70 (56) |  |
| Lower lung zone No | 53 (68.8) | 86 (68.8) | 1 |
| Lower lung zone Yes | 24 (31.2) | 39 (31.2) |  |
| Pleural No | 62 (80.5) | 90 (72.0) | 0.184 |
| Pleural Yes | 15 (19.5) | 35 (28) |  |
| Miliary No | 70 (90.9) | 117 (93.6) | 0.583 |
| Miliary Yes | 7 (9.1) | 8 (6.4) |  |
| Cavity No | 54 (70.1) | 89 (71.2) | 0.875 |
| Cavity Yes | 23 (29.9) | 36 (28.8) |  |
| **Abnormal CT chest N (%)** | | | |
| Tree-in-bud sign No | 57 (74) | 90 (72) | 0.871 |
| Tree-in-bud sign Yes | 20 (26) | 35 (28) |  |
| Segmental/lobar consolidation No | 42 (54.5) | 63 (50.4) | 0.664 |
| Segmental/lobar consolidation Yes | 35 (45.5) | 62 (49.6) |  |
| Cavity lesion No | 41 (53.2) | 72 (57.6) | 0.562 |
| Cavity lesion Yes | 36 (46.8) | 53 (42.4) |  |
| Lymphadenopathy No | 59 (76.6) | 108 (86.4) | 0.086 |
| Lymphadenopathy Yes | 18 (23.4) | 17 (13.6) |  |
| Miliary lesion No | 62 (80.5) | 109 (87.2) | 0.230 |
| Miliary lesion Yes | 15 (19.5) | 16 (12.8) |  |
| Calcified granuloma No | 61 (79.2) | 106 (84.8) | 0.341 |
| Calcified granuloma Yes | 16 (20.8) | 19 (15.2) |  |
| **Oxygen requirement N (%)** | | | |
| NC | 61 (79.2) | 106 (84.8) | 0.448 |
| NC (Yes) | 16 (20.8) | 19 (15.2) |  |
| FM (No) | 60 (77.9) | 88 (70.4) | 0.257 |
| FM (Yes) | 17 (22.1) | 37 (29.6) |  |
| TUBE (No) | 65 (84.4) | 117 (93.6) | 0.05 |
| TUBE (Yes) | 12 (15.6) | 8 (6.4) |  |
| **Complications** |  |  |  |
| Liver injury No | 70 (90.9) | 115 (92) | 0.799 |
| Liver injury Yes | 7 (9.1) | 10 (8) |  |
| N/V No | 73 (94.8) | 113 (90.4) | 0.298 |
| N/V Yes | 4 (5.2) | 12 (9.6) |  |
| AKI No | 74 (96.1) | 117 (93.6) | 0.538 |
| AKI Yes | 3 (3.9) | 8 (6.4) |  |
| Cutaneous reaction No | 76 (98.7) | 124 (99.2) | 1 |
| Cutaneous reaction Yes | 1 (1.3) | 1 (0.8) |  |
| **Outcomes N (%)** | | | |
| Cured at 6 months No | 71 (92.2) | 119 (95.2) | 0.541 |
| Cured at 6 months Yes | 6 (7.8) | 6 (4.8) |  |
| Cured at 9 months No | 75 (97.4) | 119 (95.2) | 0.713 |
| Cured at 9 months Yes | 2 (2.6) | 6 (4.8) |  |
| Cured longer duration No | 69 (89.6) | 115 (92) | 0.627 |
| Cured longer duration Yes | 8 (10.4) | 10 (8) |  |
